# Clinical and radiographic outcomes following open Latarjet with single button technique

**DOI:** 10.1016/j.xrrt.2026.100763

**Published:** 2026-05-05

**Authors:** José Andrés Ruiz, Rodrigo López, Luis Alfredo Moreno, Luis Felipe Herrera, Sofía Muñoz-Medina, Hernando Canal Torres

**Affiliations:** a Department of Orthopedics and Traumatology, Shoulder and Elbow Surgery, Clínica Reina Sofía, Bogotá, Colombia; b Specialization Program in Orthopedics and Traumatology, Unisanitas, Bogotá, Colombia; c Research Unit, Unisanitas, Bogotá, Colombia; d Department of Orthopedics and Traumatology, Shoulder and Elbow Surgery, Clínica Universitaria Colombia, Bogotá, Colombia

**Keywords:** Latarjet, Button, Shoulder, Shoulder dislocation, Coracoid process, Glenoid cavity

## Abstract

**Background:**

Anterior shoulder instability with significant glenoid bone loss remains a therapeutic challenge. The Latarjet procedure is effective, but screw fixation is associated with complications. Single-button fixation may provide comparable clinical and radiological outcomes with fewer complications, particularly in open approaches.

**Methods:**

A retrospective study was conducted at a level IV university hospital. Patients older than 18 years with anterior instability undergoing their first open Latarjet with single-button fixation between June 2023 and June 2024 were included. Demographic, clinical, imaging, and functional variables were assessed. Bone healing was evaluated with 3D computed tomography at 1 year, and functional outcomes were measured using the Disabilities of the Arm, Shoulder and Hand, Western Ontario Shoulder Instability Index, and visual analog scale for pain.

**Results:**

Eight patients (87.5% male; mean age 32 ± 4.7 years; follow-up 13.8 ± 1.6 months) were included. No recurrences or post-operative complications were reported. Complete bone healing occurred in 37.5%, partial in 37.5%, and absent in 25%. Final visual analog scale was 1.0 ± 0.64, Disabilities of the Arm, Shoulder and Hand 9.7 ± 6.6, and Western Ontario Shoulder Instability Index 42.4 ± 11.2. The apprehension sign resolved in all cases.

**Conclusion:**

Open Latarjet using single-button fixation with conjoint tendon augmentation demonstrated favorable clinical and functional outcomes, with no implant-related complications or clinical recurrences at a minimum 1-year follow-up. Although the rate of complete bone healing was lower compared to arthroscopic techniques with double-button fixation and double screws, this simplified approach appears to be safe and reproducible, reducing technical complexity, costs, and potential neurovascular risks

Anterior shoulder instability (ASI) with significant glenoid bone loss is a common and challenging condition, particularly in young and active patients. It is estimated that up to 90% of patients with recurrent instability present with substantial glenoid bone defects, which limit the success of soft tissue–only stabilization procedures such as Bankart repair or remplissage.^9^^,^^13^ In these cases, bone augmentation techniques have shown superior outcomes, with the Latarjet procedure being one of the most widely used techniques for restoring glenohumeral stability. Since its first description by Michel Latarjet in 1954, the procedure has been a standard for many surgeons due to its so-called “triple effect”: restoration of glenoid bone stock, dynamic stabilization provided by the conjoint tendon, and capsulolabral repair.^2^^,^^15^

Indications for the Latarjet procedure mainly include recurrent anterior instability with glenoid bone loss greater than 15%-25%, “engaging” Hill-Sachs lesions, and failed soft-tissue stabilization procedures such as Bankart repair.^4^^,^^5^^,^^25^ Both open and arthroscopic approaches have shown low recurrence rates and high return to sports activity, although complications remain, particularly those related to graft fixation. Traditionally, screw fixation has been the gold standard; however, it has been associated with significant complication rates, including hardware irritation, implant migration or breakage, and the need for reoperation.^1^^,^^6^^,^^7^ Over the past decade, alternative fixation methods such as suture-button systems have been proposed, aiming to achieve stable fixation with a potentially lower complication profile.^5^^,^^8^

Despite these advances, there is still a lack of evidence regarding the clinical and radiological outcomes of suture-button fixation in open Latarjet procedures. Most published studies have focused on arthroscopic techniques, leaving open approaches underexplored in terms of bone healing, recurrence of instability, and functional outcomes. This study aimed to describe these outcomes in patients undergoing open Latarjet with single-button fixation, hypothesizing that this technique may provide comparable bone healing and functional results with a lower rate of implant-related complications compared to screw fixation. Moreover, the present study seeks to furnish evidence concerning the feasibility of this technique within contexts where the open approach remains the prevailing standard of care.

## Methods

A retrospective observational study was conducted at a level IV university hospital. Adults over 18 year old diagnosed with ASI who underwent their first surgical procedure for glenohumeral stabilization, performed as an open Latarjet with single-button fixation, between June 2023 and June 2024 were included. The patients younger than 18 year old are usually treated in the first episode and manage with soft-tissue procedures. All procedures were performed by a single experienced shoulder surgeon using a standardized technique, and a minimum clinical and radiological follow-up of 1 year was required. Inclusion criteria were age ≥18 years, anterior glenohumeral instability with significant glenoid bone loss measured as >15% of the glenoid in the pre-operative 3D computed tomography (CT) scan using the perfect circle technique, and first surgical procedure for glenohumeral stabilization. Exclusion criteria included missing data in medical or surgical records, previous surgical stabilization for glenohumeral instability, arthroscopic approach, double-button fixation, calcium metabolic disorders, or neurovascular disorders of the upper limb.

All surgeries were performed under general anesthesia with the patient in a beach-chair position with 10° side inclination. A deltopectoral approach was used through an incision along the anterior axillary fold, directed approximately 6 cm over the lateral border of the conjoint tendon in direction of the coracoid. Layered dissection was performed to identify the coracoid process, followed by tenotomy of the pectoralis minor and release of the coracoacromial ligament to expose the coracoid base. Osteotomy was performed using a 90° oscillating saw with medial-to-lateral orientation under neurovascular protection. The undersurface of the coracoid graft was decorticated to obtain a congruent bone-to-bone contact surface. Through a window in the middle third of the subscapularis, a T-shaped capsulotomy was performed to expose and prepare the glenoid defect with a saw. Using a dedicated guide for button fixation, a tunnel was drilled through the center of the coracoid graft, which was positioned at the inferior glenoid rim (5-o'clock position for right shoulders, 7-o'clock for left shoulders) and passed to the posterior glenoid rim. Fixation was achieved with a single-button system (ULTRABUTTON; Smith & Nephew, Andover, MA, USA),^24^ tensioned posteriorly up to 100 N in 2 stages using the Nice knot technique. Additional stabilization was achieved by augmenting the inferior portion of the conjoint tendon with a knotless anchor secured to the inferior rim of the glenoid. Articular congruency was verified, and closure was performed in layers with postoperative immobilization in a sling. In none of the cases were additional procedures performed.

Variables collected included demographic and clinical data (sex, age, operated shoulder side, pre-operative elevation, external and internal rotation, American Society of Anesthesiologists classification, comorbidities, age at first instability episode, and number of prior dislocations), radiological findings (Hill-Sachs lesions, bony Bankart, rotator cuff involvement, and percentage of pre-operative glenoid bone loss), and intraoperative data (complications, bleeding, surgical time). Post-operative outcomes included recurrence of instability, defined as documented episodes of dislocation or subluxation during follow-up, range of motion at the last follow-up (>1 year), post-operative complications, bone healing of the coracoid graft evaluated using 3D CT at 1 year and evaluated for 2 authors who were blinded to the rest of the variables, pain level measured by the visual analog scale (VAS), and functional outcomes assessed with the Disabilities of the Arm, Shoulder and Hand (DASH) and the Western Ontario Shoulder Instability Index (WOSI) administered after 12 months.

Statistical analysis was performed using Stata 17 (StataCorp, College Station, TX, USA). Quantitative variables were described as means and standard deviations or medians and interquartile ranges according to distribution assessed by the Shapiro–Wilk test. Qualitative variables were expressed as absolute and relative frequencies. Bivariate analysis according to bone healing status was conducted using Student *t*-test or the Mann–Whitney *U* test for continuous variables and the chi-square or Fisher exact test for categorical variables. A *P* value <.05 was considered statistically significant.

## Results

Eight patients (7 men and 1 woman) with a mean age of 32.0 ± 4.7 years (range, 24-39 years) were included. The operated shoulder was predominantly the right side (87.5%), and American Society of Anesthesiologists classification was I in six patients (75%) and II in 2 patients (25%). The clinical apprehension sign was positive in 100% of patients pre-operatively. Two patients had relevant comorbidities (hypertension, diabetes mellitus, and smoking), and although no statistically significant associations were observed, it is noteworthy that patients with diabetes mellitus or smoking history did not achieve complete bone healing (Table I).

**Table I:** Demographic variables and pre-operative imaging findings.

Variable	n (%)/mean ± SD
Age (yr)	32.0 ± 4.7
Sex	
Male	7 (87.5)
Female	1 (12.5)
Operated side	
Right	7 (87.5)
Left	1 (12.5)
ASA classification	
ASA I	6 (75)
ASA II	2 (25)
Comorbidities	
None	5 (62.5)
Hypertension	1 (12.5)
Smoking	1 (12.5)
Diabetes mellitus	1 (12.5)
Number of previous dislocations	6.9 ± 3.2
Age at first instability episode (yr)	22.8 ± 5.3
Bony Bankart lesion	8 (100)
Hill-Sachs lesion	8 (100)
Rotator cuff tear	0 (0)
Glenoid bone loss (%)	26.2 ± 7.1

*ASA*, American Society of Anesthesiologists; *SD*, standard deviation.

Pre-operative imaging demonstrated a mean glenoid bone loss of 26.2% ± 7.1% (range, 18.9%-39.6%). All patients presented with bony Bankart and Hill-Sachs lesions, with no evidence of rotator cuff tears. The mean number of previous dislocations was 6.9 ± 3.2, and the mean age at the first instability episode was 22.8 ± 5.3 years. The mean time from the initial instability episode to surgical intervention was 9.5 ± 6.4 years. All patients underwent pre-operative CT evaluation, and 4 had additional magnetic resonance imaging assessment.

Intraoperatively, bleeding was minimal to mild, ranging from 10 to 100 ml, and the mean surgical time was 75.8 ± 1.46 minutes. Clinical follow-up averaged 13.8 ± 1.6 months. No clinical recurrences of instability or post-operative complications were recorded at more than 1 year of follow-up. Pain outcomes at the end of follow-up showed a mean VAS score of 1.0 ± 0.64, reflecting satisfactory pain control in all cases.

**Figure 1:**
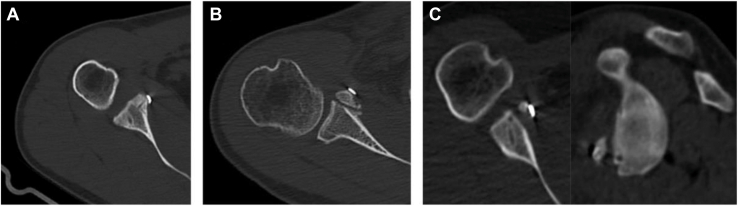
Post-operative CT scan. (**A**) Complete healing. (**B**) Partial healing. (**C**) No healing. *CT*, computed tomography.

**Figure 2:**
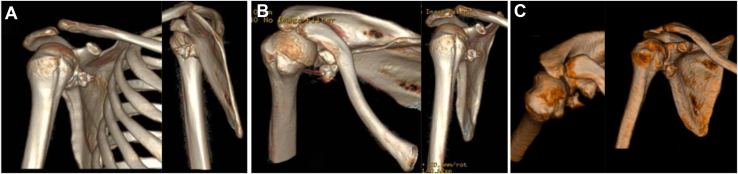
Post-operative 3D-CT scan. (**A**) Complete healing. (**B**) Partial healing. (**C**) No healing. *3D*, three-dimensional; *CT*, computed tomography.

Radiological evaluation with 3D CT demonstrated complete graft healing in 3 patients (37.5%), partial healing in 3 patients (37.5%), and no evidence of healing in 2 patients (25%). Mean radiological follow-up time was 13.4 ± 1.6 months (Figs. 1 and 2).

Functional outcomes improved globally. The mean DASH score was 9.7 ± 6.6, and the mean WOSI score was 42.4 ± 11.2. Active elevation improved from a pre-operative mean of 140.0° ± 25.3 to 151.3° ± 17.5 post-operatively. External rotation improved from 53.8° ± 8.3 to 56.3° ± 8.0, whereas internal rotation remained unchanged, with most patients maintaining the T10 vertebral level. The apprehension sign disappeared in all patients.

When analyzing the relationship between bone healing and intraoperative variables (Table II), patients without healing tended to have greater intraoperative bleeding (median 60 ml vs. 20 ml in complete healing) and longer surgical times (82 ± 9.8 min vs. 64 ± 5.2 min), although these differences were not statistically significant (*P* = .312 and *P* = .292, respectively). Functional outcomes varied between groups. Patients with complete healing demonstrated better WOSI values (mean 35.6 vs. 49); however, DASH scores did not show the same pattern (median 9.2 vs. 7.1). None of these differences reached statistical significance (*P* > .3). Range-of-motion analysis according to healing status showed no significant differences, although a slight reduction in external rotation was observed in patients with complete healing (45° vs. 51°; *P* = .27), (Table III).

**Table II:** Comparison of variables according to bone healing status.

Variable	Complete healing n (%)	Partial healing n (%)	No healing n (%)	*P* value
Age (yr), mean ± SD	34.0 ± 4.6	31.3 ± 6.3	30.0 ± 1.4	.481
Sex				.386
Male	2 (66.7)	3 (100)	2 (100)	
Female	1 (33.3)	0 (0)	0 (0)	
Operated side				.386
Right	3 (100)	2 (66.7)	2 (100)	
Left	0 (0)	1 (33.3)	0 (0)	
ASA classification				.641
ASA I	2 (66.7)	2 (66.7)	2 (100)	
ASA II	1 (33.3)	1 (33.3)	0 (0)	
Comorbidities				.380
Diabetes mellitus	0 (0)	1 (33.3)	0 (0)	
Hypertension	1 (33.3)	0 (0)	0 (0)	
Smoking	0 (0)	0 (0)	1 (50.0)	
Glenoid bone loss (%), mean ± SD	25.3 ± 6.5	26.0 ± 11.8	28.8 ± 3.2	.485
Previous dislocations, mean ± SD	5.3 ± 3.1	8.3 ± 4.7	7.0 ± 2.8	.815
Age at first instability (yr), mean ± SD	29.7 ± 1.5	18.0 ± 0.1	18.5 ± 5.0	.238
Pre-operative MRI	1 (33.3)	1 (33.3)	2 (100)	.264
Surgical time (min), mean ± SD	64.0 ± 5.3	83.3 ± 20.8	82.0 ± 9.9	.292
Intraoperative bleeding (ml), median (IQR)	20 (10-50)	20 (20-20)	60 (20-100)	.312
Pre-operative elevation (°), mean ± SD	156.7 ± 15.3	126.7 ± 32.2	155.0 ± 7.1	.377
Pre-operative external rotation (°), mean ± SD	60.0 ± 10.0	53.3 ± 5.8	45.0 ± 7.1	.792
Pre-operative internal rotation				.438
T10	1 (33.3)	2 (66.7)	0 (0)	
T12	1 (33.3)	0 (0)	0 (0)	
T7	1 (33.3)	0 (0)	1 (50.0)	
Post-operative elevation (°), median (IQR)	160 (120-170)	150 (130-160)	160 (160-160)	.497
Post-operative external rotation (°), mean ± SD	45.0 ± 5.0	51.7 ± 7.6	50.0 ± 14.1	.539
Post-operative internal rotation				.276
T10	3 (100)	2 (66.7)	1 (50.0)	
T12	0 (0)	1 (33.3)	0 (0)	
T7	0 (0)	0 (0)	1 (50.0)	
DASH score, median (IQR)	9.2 (5-12.5)	6.7 (5-24.2)	7.1 (5-9.2)	.330
WOSI score, mean ± SD	35.7 ± 9.5	44.7 ± 5.7	49.0 ± 18.4	.461
Radiological follow-up (mo), mean ± SD	13.7 ± 1.5	12.0 ± 0.1	14.5 ± 3.5	.393
Clinical follow-up (mo), mean ± SD	14.3 ± 2.1	14.0 ± 1.7	12.5 ± 0.7	.652
Final VAS, mean ± SD	0.0 ± 0.0	1.0 ± 0.0	2.0 ± 0.0	.180

*ASA*, American Society of Anesthesiologists; *VAS*, visual analog scale; MRI, magnetic resoannce imaging; *DASH*, Disabilities of the Arm, Shoulder and Hand; *WOSI*, Western Ontario Shoulder Instability Index; *SD*, standard deviation; *IQR*, interquartile range.

**Table III:** Pre-operative and post-operative range of motion and apprehension sign.

Variable	Pre-operative (mean ± SD/n)	Post-operative (mean ± SD/n)
Active elevation (°)	140.0 ± 25.3	151.3 ± 17.5
External rotation (°)	53.8 ± 8.3	56.3 ± 8.0
Internal rotation (most frequent vertebral level)	T10	T10
Clinical apprehension sign	8/8 (100% positive)	0/8 (0% positive)

*SD*, standard deviation.

## Discussion

The Latarjet procedure is a widely accepted technique for the treatment of ASI, especially in patients with significant glenoid bone loss and recurrence after previous stabilization procedures.^11^^,^^12^ This is a technically demanding procedure in which correct implant positioning and adequate fixation to the glenoid neck are key to avoiding associated complications.^5^^,^^13^^,^^14^ Multiple variations have been made to the original technique since its description by Michel Latarjet in 1954,^15^ including the intra- or extra-articular position of the graft, which may reduce post-operative osteoarthritis^16^^,^^17^; bone-block fixation techniques with or without capsulolabral structure repair^18^^,^^19^; and arthroscopically assisted surgery.^8^^,^^20^ Traditional screw fixation presents a considerable complication rate, which has motivated the development of alternatives such as suture-button systems.^3^^,^^26^

**Figure 3:**
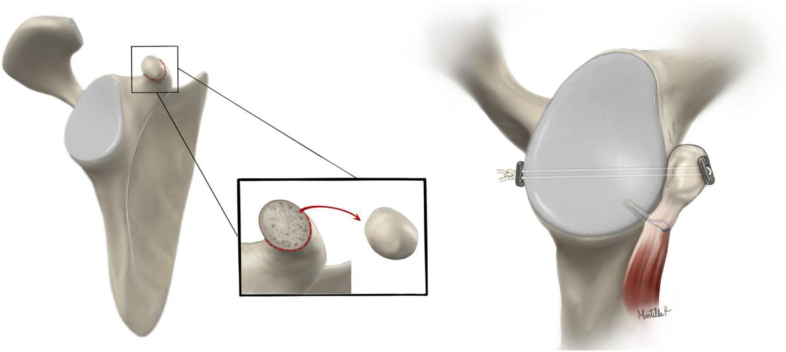
The coracoid process is transferred, passed through the subscapularis, and fixed on the anterior neck of the scapula with 1 cortical button, and then the conjoint tendon is fixed to the inferior border of the glenoid with a suture and a knotless anchor.

The present study evaluated the clinical, functional, and radiological outcomes of adult patients with ASI treated between June 2023 and June 2024 in a level IV university hospital with a novel variation of the Latarjet technique. This modification consisted of performing the procedure completely open, with single-button fixation associated with augmentation using an anchor securing the conjoint tendon to the glenoid, providing a bumper effect on the inferior edge of the anterior glenoid and additional rotational stabilization of the graft. Complete healing was observed in 37.5% of cases and partial healing in another 37.5%, with no implant-related complications. Although these rates are lower than those reported in arthroscopic techniques with double-button fixation, such as Boileau et al,^4^ who documented a 90% healing rate in a series of 32 patients undergoing arthroscopic Latarjet with double-button fixation, and Xu et al,^28^ who reported 100% healing in 102 patients treated with arthroscopic Latarjet using double-button fixation, it should be emphasized that this study used a single implant and a completely open approach that could change the environment of the soft-tissues (Fig. 3.). This approach reduces technical complexity, procedural costs, and neurovascular risks inherent to the arthroscopic technique.^22^^,^^23^ To date, in our review, only Erickson et al^10^ described a single button open approach, which shows improvement in American Shoulder and Elbow Surgeons, VAS, and Single Assessment Numeric Evaluation scores. However, Wang et al^27^ described an open approach with arthroscopic assistance and single-button fixation in six patients compared with six patients treated with the traditional technique; all achieved healing, although one patient in the button group developed graft osteolysis outside the “perfect circle” of the glenoid, and 1 patient in the screw group had complete graft osteolysis. Similarly, Nair et al^22^ reported 70% bone healing in patients treated with a single implant (FiberTape), a result comparable to the partial and complete healing rates observed in this study.

Regarding functional outcomes, the results were comparable to studies such as Xu et al,^28^ who reported improvements in Rowe (94.5), American Shoulder and Elbow Surgeons (95.2), and Walch-Duplay (95.6) scores. In this study, patients achieved a mean DASH score of 9.2 and a WOSI score of 40 points, along with the disappearance of the clinical apprehension sign in 100% of cases, reflecting clinically relevant improvement, although the same assessment tools were not used. In addition, a reduction in pain was observed (VAS: 3.3 to 1.1),^21^ similar to the post-operative pain reported in this study. Several systematic reviews have shown no significant differences in functional outcomes between open and arthroscopic techniques, such as the one conducted by Hurley in 2019, although it showed a trend toward higher revision rates in arthroscopic techniques (2.4% vs. 5.4%, *P* = .06) and superiority of the open technique in reducing post-operative apprehension (10.2% vs. 35.7%; *P* = .05).^14^

In terms of complications, Boileau et al^5^ reported a 3.3% complication rate (hematoma and transient neurological irritation), and Xu et al^28^ reported 1.9% (post-traumatic redislocation and reversible stiffness), with no infections or implant failures. No perioperative complications or implant failures were recorded in our cohort. This low-risk profile is consistent with Rosenow et al,^23^ who proposed the button as a safe biomechanical alternative to screws, and with Manfredi et al,^18^ who concluded in a systematic review that the button provides resistance comparable to screw fixation, although with greater micromobility in biomechanical studies, which may favor bone remodeling and a lower rate of complications due to implant prominence or breakage.

When comparing range of motion with healing status, patients with complete healing tended to lose a few degrees of external rotation, although this was not significant in functional scores. Similar results were reported by Zhu et al,^29^ who evaluated mobility in open and arthroscopic groups and found a nonsignificant reduction in post-operative external rotation (from 56.7° to 50.4° in the arthroscopic group and from 58.6° to 54.1° in the open group), without assessing associations with healing status.

Although the sample size was insufficient to establish strong associations, it is noteworthy that patients with diabetes mellitus and a history of smoking did not achieve complete healing, which is consistent with reports in the literature. Dalmas et al^8^ demonstrated that smoking increased the risk of nonunion at three months after surgery by more than 12 times (adjusted odds ratio = 12.17; *P* = .001), while diabetes, although not directly identified as a risk factor in studies on this technique, has been associated with nonunion in other orthopedic procedures, such as lumbar and foot arthrodesis.^3^

An important difference between the patients in this study and those in previous studies is the use of a single button and an associated anchor for the conjoint tendon in the traditional open approach, compared with the arthroscopic double-button technique or reinforcements with cerclage or additional screws. Although scarcely described in the literature, this technique has proven to be safe and reproducible. Gutiérrez-Zúñiga et al^11^ proposed a similar approach in a technical note, although no clinical reports have been published yet. This technical simplification appears safe in our setting, although the partial healing observed in a significant proportion suggests that fixation optimization may be necessary, and long-term follow-up is required to evaluate potential associated complications.

This study has several limitations that must be considered when interpreting the results. First, its retrospective design may introduce selection bias and limits the ability to establish causal relationships. In addition, the sample size was small, with only 8 patients, which limits the generalizability of the findings. The low number of cases was not due to clinical criteria but rather to restrictions imposed by recent changes in insurance and surgical authorization policies within the health care system, which limited access to this technique during the study period. There was also no comparative control group (eg, screw or double-button fixation) to evaluate direct differences in outcomes. Finally, although clinical and tomographic follow-up exceeded 1 year, this period may be insufficient to evaluate late outcomes, such as arthropathy development or progressive graft resorption.

Despite these limitations, the study has important strengths. The surgical technique was standardized and performed by a single experienced surgeon, reducing operative variability. A rigorous clinical (DASH, WOSI, and VAS) and radiological (3D CT) evaluation protocol was employed, providing an objective and detailed characterization of outcomes. To our knowledge, this is the first series to report clinical and radiological outcomes of the Latarjet technique with single-button fixation and rotational augmentation with an anchor, contributing evidence on its feasibility, safety, and early results.

## Conclusion

Open Latarjet using single-button fixation with conjoint tendon augmentation demonstrated favorable clinical and functional outcomes, with no implant-related complications or clinical recurrences at a minimum 1-year follow-up. Although the rate of complete bone healing was lower compared to arthroscopic and open techniques with double-button fixation, this simplified approach appears to be safe and reproducible, reducing technical complexity, costs, and potential neurovascular risks. Partial healing did not translate into clinical instability or poorer functional outcomes in the short term; however, long-term studies are required to determine its impact on graft behavior and potential late complications. This technique represents a viable alternative in selected cases, especially in settings where resource optimization and technical feasibility are relevant considerations.

## Disclaimers:

Funding: No funding was disclosed by the authors.

Conflicts of interest: The authors, their immediate families, and any research foundations with which they are affiliated have not received any financial payments or other benefits from any commercial entity related to the subject of this article.
